# Accuracy of self-perceived cardiovascular disease risk and factors predicting risk underestimation in perimenopausal and postmenopausal women in Ismailia, Egypt

**DOI:** 10.1186/s42506-024-00170-y

**Published:** 2024-10-01

**Authors:** Mirella Youssef Tawfik, Hanan H. Soliman, Zeinab F. Abdel-Fatah

**Affiliations:** Department of Public Health, Occupational and Environmental Medicine, Faculty of Medicine, Suez Canal University, Ismailia, Egypt

**Keywords:** Self-perceived CVD risk, Predicted CVD risk, CVD risk underestimation, Perimenopausal women, Postmenopausal women, Health literacy

## Abstract

**Background:**

Cardiovascular disease (CVD) is the leading cause of death globally, with women at higher risk after menopause. This increased risk is attributed to both aging and hormonal changes. Prior research has established a link between CVD risk perception and adopting healthy behaviors to prevent CVD. This study aimed to assess the accuracy of self-perceived CVD risk in perimenopausal and postmenopausal women, and to identify factors that predict CVD risk underestimation among them.

**Methods:**

A cross-sectional study was conducted in the administrative sectors of Suez Canal University campus in Ismailia, Egypt, over a period of eight months starting in July 2022. A total of 390 eligible women (employees and workers) were randomly selected. Participants were interviewed to obtain data on demographics, medical history, self-perceived risk of CVD, self-perceived general health, awareness of factors that increase the risk of developing CVD, perceived stress, health literacy, numeracy, and self-perceived 10-year risk of developing major cardiovascular events. They also underwent measurements of blood pressure, weight, and height. The updated 2019 WHO/CVD risk non-laboratory-based prediction chart for the North Africa and Middle East Region was used to predict the 10-year risk of major cardiovascular events for the study participants. Risk accuracy was measured by comparing self-perceived CVD risk with predicted CVD risk.

**Results:**

The ratio of self-perceived to predicted moderate/high CVD risk was 27.7% to 44.3%, respectively. The accuracy of CVD risk perception was 68.2%. Kappa analysis results showed fair and significant agreement between self-perceived and predicted CVD risk (kappa ± SE = 35.9 ± 4.1%, *p* < 0 .001). The proportion of women who underestimated their risks was 24.1%. Of those in the high-risk group, 93.3% underestimated their CVD risk, compared to 50.6% in the moderate-risk group. Factors that significantly predicted CVD risk underestimation included being married (aOR 14.5; 95% CI 1.4-149.9), low income (aOR 2.321; 95% CI 1.09-4.909), high BMI (aOR 4.78; 95% CI 1.9-11.9), hypertension (aOR 3.5; 95% CI 2-6.2), and old age (aOR 1.46; 95% CI 1.3-1.6).

**Conclusions:**

Approximately one-third of our study participants misperceived their CVD risk; of those who did, 75.8% underestimated it. Marital status, old age, low income, high BMI, and hypertension strongly predicted CVD risk underestimation. These findings identified the menopausal women subgroups that could benefit from targeted health interventions designed to reduce CVD risk underestimation and improve risk accuracy.

## Introduction

Cardiovascular disease (CVD) represents the top cause of death worldwide, with ischemic heart disease and stroke contributing to more than 80% of these deaths [1]. In 2019, about 29% of women worldwide and 41% of women at the country level in Egypt died due to ischemic heart disease and stroke [2]. Cardiovascular disease risks, such as visceral adiposity, dyslipidemia, glucose intolerance, and hypertension, are more prevalent in women at the time of menopause compared with premenopausal status [3]. This rise in CVD risks is likely related to a combination of aging and the decline in cardio-protective female sex hormones, especially estrogens, characteristic of the menopausal transition [4].

Framingham researchers reported in 1976 that age-matched postmenopausal women had a 2.6-fold higher incidence of cardiovascular events than premenopausal women [5]. Furthermore, women experience a7–10-year latency in the onset of CVD in comparison to men, a period that corresponds with the menopause transition [6]. Additionally, observational studies have consistently demonstrated a positive correlation between ischemic heart disease and early menopause age [7–9]. Therefore, it has been proposed that the midlife withdrawal of endogenous sex steroids linked to the menopausal transition is responsible for the higher risk of ischemic heart disease in older women [10].

Guidelines on CVD prevention recommend the use of CVD risk assessment tools to screen those at higher risk of CVD and provide them with early preventive measures [11]. The most important measure to prevent CVD is to promote healthy lifestyle behaviors throughout life [12]. Risk perception is considered a core psychological construct that impacts change and the maintenance of an individual's health behavior [13]. Although a correct CVD risk perception is critical for adopting healthy lifestyle behaviors and adhering to medical treatments [14], individuals often underestimate their risk of developing CVD [15].

There is limited knowledge about the accuracy of perceived CVD risk among perimenopausal and postmenopausal women. A study in Malaysia found that approximately half of the women in this group underestimated their CVD risk. The study also identified that older age, high systolic blood pressure, and diabetes were factors associated with this underestimation [16].

A study conducted in Iran showed a significant positive relationship between health literacy (HL) and the perceived risk of CVD in middle-aged Iranian women [17]. This suggests that women with lower HL levels may be more likely to underestimate their CVD risk. Another study conducted on a Swedish population sample of both genders examined the association of a comprehensive range of factors, including perceived stress, perceived general health, HL, self-perceived CVD risk factor knowledge, and numeracy skills, in addition to demographic and medical factors, with perceived CVD risk. While this previous study provided valuable insights into individuals' risk perceptions, it did not assess CVD risk underestimation [18].

Studying the accuracy of self-perceived CVD risk among perimenopausal and postmenopausal women at the community level will provide a broader understanding of the general population’s situation. To our knowledge, there have been no studies in Egypt specifically examining CVD risk perception among perimenopausal and postmenopausal women. Identifying factors that predict CVD risk underestimation will help pinpoint subgroups needing targeted health interventions. This study aims to assess the accuracy of self-perceived CVD risk in perimenopausal and postmenopausal women and identify predictors of CVD risk underestimation in this group.

## Methods

### Study design, setting and subjects

This cross-sectional study was carried out in the administrative sectors of the Suez Canal University campus in Ismailia, Egypt, from July 2022 to February 2023. Women employees and workers aged ≥ 45 years working in the administration offices of the university and faculties who had fulfilled the criteria of the target population of perimenopausal, and postmenopausal women were recruited. The perimenopausal group included women who had experienced persistent cycle irregularity, with a difference in cycle length of at least 7 days on two or more occasions within the previous 10 cycles, and/or amenorrhea for at least 60 days but no more than 12 months within the last year. The postmenopausal group included women who had experienced their final menstrual period more than one year prior [19]. Exclusion criteria included the following: having undergone the removal of one or both ovaries or a hysterectomy; having polycystic ovary syndrome or premature ovarian failure; experiencing premature menopause (defined as cessation of menstrual periods before the age of 40); using exogenous hormones; experiencing amenorrhea following cancer treatment; confirmed pregnancy; and a history of CVD, defined as being diagnosed with or treated for CVD.

### Sample size

**Fig. 1:**
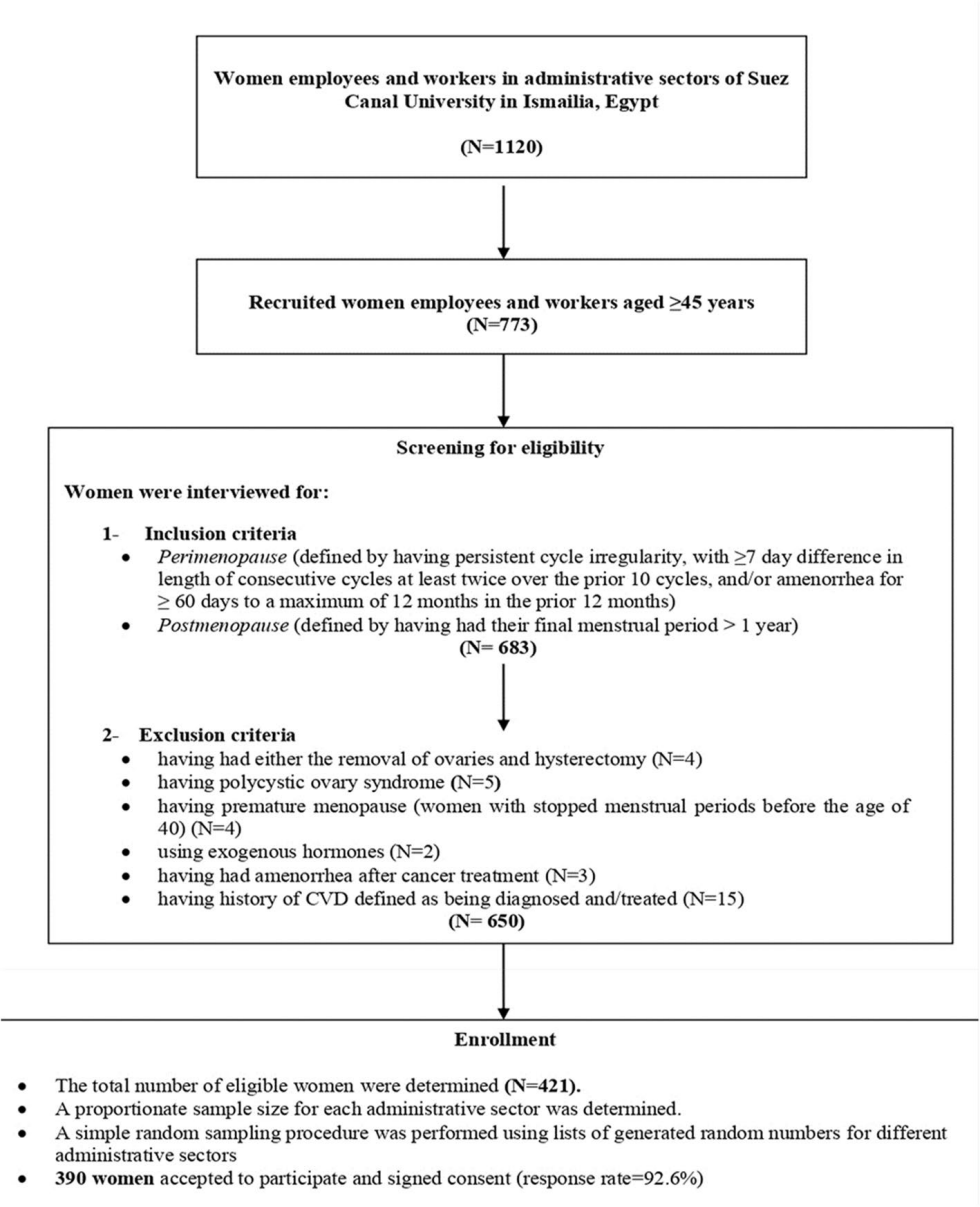
Flow diagram of study participant enrollment

The sample size was calculated using the G*Power program for Windows [20]. Using linear multiple regression (fixed model R2 deviation from zero), a total calculated sample of 366 was estimated to be enough for testing the strength of association between potential predicting variables and underestimation of perceived CVD risk among perimenopausal and postmenopausal women. This calculation was based on a multiple linear regression model involving at least 20 predictors, at effect size f^2^ = 0.06, α error = 0.05, and power = 0.80. This number was increased to 421 to allow for an expected non-response rate of 15%. The sample size of participants from each administrative sector was determined based on the proportionate size of the eligible female population in each sector. Using a random integer generator from a reputable software program (RANDOM.ORG's integer generator), women were randomly selected from a list of coded-eligible participants and invited to participate in the study. The study’s aims and procedures were explained to the women. Those who accepted to participate signed consent forms, resulting in a final total of 390 participants and a response rate of 92.6% (Fig. 1).

### Data collection tools

Participants’ self-reported data were collected using a structured interview questionnaire composed of two parts: the first part comprises the socio-demographics data including age, education, marital status, monthly income, and occupation and medical history data of study participants. Smoking status, experiencing chest pain, and shortening of breath were assessed using one question for each with a dichotomous response (Yes or No). A “smoker” is referred to as someone who smokes daily or occasionally, and a non-smoker is referred to as someone who has stopped smoking or has never smoked. Experiencing chest pain refers to feeling tingling or pain in the chest, while shortness of breath refers to difficulty breathing, particularly when going upstairs or walking quickly. Having diabetes, hypertension, or hyperlipidemia was defined as being diagnosed and/or treated.

The second part comprises the participants’ self-perceived risk of developing CVD (myocardial infarction and stroke) in the next 10 years, their self-perception regarding their general health, and their awareness of factors that can increase the risk of developing CVD. The self-perceived risk of developing CVD in the next 10 years was assessed using a categorical measure with a 3-point Likert scale: low, moderate, and high degree of risk, using the question “How do you perceive your risk of having a myocardial infarction or stroke in the next ten years?’’. Participants were also asked to rate their current health status on a scale from 0 to 10 with 5 equal intervals, rated as very poor, poor, fair, good, and very good [18]. Awareness of factors that can increase the risk of experiencing CVD was assessed using ten items. These items included obesity, diabetes, hypertension, hyperlipidemia, aging/menopause, hereditary factors and behavior related factors as unhealthy diet, lack of exercise, smoking, and drinking alcohol which were considered to increase the risk of developing CVD [4, 21]. Responses were dichotomized into “Yes” (1) or “No” (0), and a total awareness score (ranging from 0-10) was obtained by summing across all items, with higher scores indicating a higher level of awareness.

Perceived stress was assessed based on its duration and intensity. To measure the perceived duration of stress, participants responded to a question which asked: "Which of these statements best represents your exposure to psychological stress during the past five years?" The scale included options ranging from short periods of stress ("never experienced stress," "experienced some periods of stress during the past five years") to moderate periods ("experienced long periods of stress that lasted one year during the past five years"), and long periods ("constant stress during the last year," "constant stress during the past five years") [18].

To evaluate the perceived intensity of stress over the previous month, the validated Arabic version of the Perceived Stress Scale (PSS-10) was used [22]. This scale consists of 10 questions with responses on a five-point Likert scale, ranging from "never" (0) to "very often" (4). The total score on the Arabic PSS-10 is calculated by summing the scores of all items. Stress levels were categorized as low (0–13), moderate (14–26), and severe (27–40), with higher scores indicating greater perceived stress. The Arabic PSS-10 has demonstrated concurrent validity and acceptable reliability [22].

Health literacy, defined as the ability to find, understand, and use health information to make informed decisions and take care of one's own health [23], was assessed using the validated Arabic 16-item short version of the European Consortium for Health Literacy Questionnaire (HLQ-EU-16) [24]. The questionnaire items address self-reported difficulties in tasks concerning decision-making in health care, disease prevention, and health promotion using a 4-point Likert scale ranging from 1 (very difficult) to 4 (very easy). Total HL scores (with a range from 0 to 16) were obtained by dichotomizing answers to 0 (very difficult/fairly difficult) and 1 (fairly easy/very easy). Scoring establishes three HL categories: 0–8 = inadequate, 9–12 = problematic, and 13–16 = sufficient HL. For the purpose of the analysis, the HL variable was dichotomized into "inadequate/problematic" and "sufficient" based on the findings of a previous study [18] and to align with determining the influence of sufficient HL compared to lower HL levels on the underestimation of CVD risk. The questionnaire had high internal consistency, and construct validity [24]. Numeracy, defined as the ability to understand and use numerical information for health decision-making [25], was assessed using the Short 3-item Version of Subjective Numeracy Scale (SNS-3), with a five-point Likert scale (with higher scores indicating high numeracy), from which a mean summary score was calculated. The SNS-3 demonstrated both high internal consistency and good evidence of criterion and construct validity [26].

Participants’ clinical data included blood pressure and body mass index (BMI) measures. The blood pressure was measured using the standard mercury sphygmomanometer in the left arm. The average of two measures (≥ 2 min apart) was obtained after the participant had been sitting for ≥ 5 min. Participants’ BMI was calculated using the formula [BMI = weight (kg) / height (m^2^)], with height and weight measured using a height measuring stand and a weighing scale, respectively. Measurements were taken with participants wearing clothing without shoes.

The 2019 World Health Organization (WHO)/CVD risk-non-laboratory-based prediction chart, tailored for the North Africa and Middle East Region, was used to estimate participants' 10-year risk of experiencing a major cardiovascular event, such as a heart attack or stroke. This chart, which follows WHO guidelines, assesses risk based on five factors: age, sex, smoking status, blood pressure, and BMI. Risk is categorized as low (less than 10%), moderate (10% to less than 20%), or high (20% or more) [27]. Participants self-perceived risk accuracy was determined by comparing their self-assessed risk of developing CVD in the next 10 years with the risk predicted by the WHO chart.

Reliability analysis of the current study sample revealed Cronbach’s alpha of 0.947 for the PSS-10, 0.963 for the HLQ-EU-16 questionnaire, and 0.708 for the SNS-3. The whole questionnaire was pre-tested on 20 women who were not included in the study. The pilot testing did not necessitate any changes to the questionnaire construction or wording. The average time required to complete the questionnaire ranged from 10 to 15 min.

### Statistical analysis

Data were assessed for normality using the Kolmogorov-Smirnov test, which indicated that continuous variables were not normally distributed. Consequently, medians and interquartile ranges (IQR) were used for descriptive statistics. Categorical variables were summarized using frequencies and percentages. The Kappa test was used to detect the degree of agreement between self-perceived and predicted CVD risk. A two-step statistical approach was employed to identify factors that predict underestimation of CVD risk. Firstly, significant variables were identified through simple logistic regression analysis (*p* < 0.05). Secondly, backward stepwise multiple logistic regression with likelihood ratios (LR) was used to create a model that included only the most relevant and statistically significant predictors. All statistical analyses were performed using SPSS 25 (Armonk, NY: IBM Corp.).

## Results

### Characteristics of study participants

The median age of study participants was 52 years with an interquartile range of 6 years, with a majority falling in the 50- < 55 age group (40.3%). Only 22.8% of study participants were in the youngest age group (45- < 50), about 40.3% of them had a university or postgraduate degree. Most participants were married (93.8%), 77.9% had a high BMI, about one-third had hypertension (34.4%), 18.2% were diabetics, and none of them were smokers. Long periods of stress and high perceived stress levels were reported in 25.6% and 37.7% of participants, respectively. The perceived state of general health was reported as “good, very good” in 81.8% of participants, HL was considered inadequate or problematic for 81.3% of the individuals (Table 1).

**Table 1:** General characteristics of a sample of women employees and workers from Suez Canal University, Ismailia, Egypt (2022–2023)

**General characteristics (*n* = 390)**	**No. (%)**
**Age (years)**	
45- < 50	89 (22.8)
50- < 55	157 (40.3)
55- < 60	144 (36.9)
**Median (IQR) **52.0 (6)	
**Educational level**	
≤ Secondary school or technical diploma	233 (59.7)
University/postgraduate degrees	157 (40.3)
**Marital status**	
Unmarried	24 (6.2)
Married	366 (93.8)
**Monthly income (LE)**	
< 5000	220 (56.4)
≥ 5000	170 (43.6)
**Occupation**	
Workers	45 (11.5)
Employees	345 (88.5)
**Menopausal status**	
Perimenopause	153 (39.2)
Post-menopause	237 (60.8)
**BMI**	
Underweight, or normal weight	86 (22.1)
Overweight, or obese	304 (77.9)
**Hypertension**	
No	256 (65.6)
Yes	134 (34.4)
**Diabetes**	
No	319 (81.8)
Yes	71 (18.2)
**High blood lipids**	
No	278 (71.3)
Yes	112 (28.7)
**Chest pain**	
No	276 (70.7)
Yes	114 (29.3)
**Shortness of breath**	
No	265 (67.9)
Yes	125 (32.1)
**Family history of CVD**	
No	331 (85.1)
Yes	59 (14.9)
**Perceived stress duration**	
Short/moderate periods of stress	290 (74.4)
Long periods of stress	100 (25.6)
**Perceived stress level**	
Low, moderate	243 (62.3)
High	147 (37.7)
**Perceived state of general health**	
Very poor, poor, fair	71 (18.2)
Good, very good	319 (81.8)
**Numeracy, median (IQR)**	2.7 (0.7)
**Awareness of CVD risk factors, median (IQR)**	7 (3)
**Health Literacy**	
Inadequate, or problematic	317 (81.3)
Sufficient	73 (18.7)

### Self-perceived CVD risk, predicted CVD risk and risk accuracy

Table 2 highlights the relationship between perceived and actual CVD risk. The comparison shows that 90.8% of those who perceived themselves as low risk were indeed at low actual risk, while 50.7% and 33.3% of those who perceived low risk were at moderate and high risk, respectively. Among those with a moderate perceived risk, 9.2% were at low actual risk, 43% were at moderate actual risk, and 60% were at high actual risk. Interestingly, none of the individuals who perceived a high risk were at low risk, but 6.3% and 6.7% were at moderate and high risk, respectively. Overall, the majority of individuals (68.2%) accurately perceived their CVD risk, while 24.1% underestimated and 7.7% overestimated their risk. The kappa statistic of 0.359 indicates a fair to moderate agreement between perceived and actual risk levels, with a statistically significant p-value (*p* = 0.000).

**Table 2:** Accuracy of participants’ CVD self-perceived risk as compared with their predicted WHO/CVD risk (*n* = 390)

**Perceived CVD risk**	**WHO/CVD risk, no. (%)**				
	**Low**	**Moderate**		**High**	**Total**
**Low**	197 (90.8)	80 (50.7)		5 (33.3)	282 (72.3)
**Moderate**	20 (9.2)	68 (43.0)		9 (60.0)	97 (24.9)
**High**	0 (0.0)	10 (6.3)		1 (6.7)	11 (2.8)
**Total No. (%)**	217 (55.7)	158 (40.5)		15 (3.8)	390 (100.0)
Kappa ± SE, *p-*value 0.359 ± 0.041, 0.000^*^					
**Risk accuracy**			**No.(%)**		
**Accurate**			266 (68.2)		
**Under-estimation**			94 (24.1)		
**Over-estimation**			30 (7.7)		

^*^Statistically significant at *p* < 0.05

### Factors that predict an underestimation of CVD risk

Factors proved significant with women’s CVD risk underestimation after simple logistic regression analysis, including age, educational level, monthly income, menopausal status, BMI, HTN, perceived stress, awareness of CVD risk factors (*p* = 0.001), shortness of breath (*p* = 0.003), family history (*p* = 0.021), and marital status (*p* = 0.045) (Table 3).

**Table 3:** Bivariate analysis of factors associated with cardiovascular risk underestimation of (*n* = 390)

**Variables**	**B**	**SE**	**Wald**	**df**	***P* value**	**Odds ratio**		**95% C.I. for EXP (B)**	
								**Lower**	**Upper**
**Age of participants**	0.276	0.031	49.404	1	0.000^*^		1.317	1.22	1.42
**Educational level** ≤ Secondary school or technical diploma^a^									
University or postgraduate degrees	0.987	0.242	16.56	1	0.000^*^		2.682	1.668	4.314
**Marital status** Unmarried^a^									
Married	2.059	1.029	4.006	1	0.045^*^		7.835	1.044	58.82
**Monthly income (LE)** ≥ 5000^a^									
< 5000	1.109	0.856	1.677	1	0.000^*^		3.030	0.56	16.22
**Menopausal status** Perimenopause^a^									
Postmenopause	1.264	0.287	19.41	1	0.000^*^		3.540	2.017	6.213
**BMI** Underweight/normal weight^a^									
Overweight/obese	1.510	0.414	13.278	1	0.000^*^		4.525	2.009	10.191
**Having hypertension** No^a^									
Yes	1.173	0.245	22.96	1	0.000^*^		3.231	2.0	5.22
**Experienced shortness of breath** No^a^									
Yes	0.729	0.245	8.886	1	0.003^*^		2.073	1.284	3.347
**Family history of CVD** No^a^									
Yes	0.700	0.304	5.314	1	0.021^*^		2.014	1.111	3.651
**Perceived stress level** Low/moderate^a^									
High	0.788	0.241	10.740	1	0.000^*^		2.200	1.373	3.525
**Awareness of CVD risk factors**	-0.203	0.061	11.211	1	0.000_*_		0.816	0.725	0.919

^a^Refers to reference group

^*^Statistically significant at *p* < 0.05

The Multivariate logistic regression analysis identifies several key factors predicting the underestimation of CVD risk. Age emerged as a significant predictor, with each additional year increasing the likelihood of underestimating CVD risk by approximately 46.4% (aOR = 1.464, *p* = 0.000). Marital status also played a role, as married individuals were significantly more likely to underestimate their risk compared to unmarried individuals, with an odds ratio of 14.531 (*p* = 0.025). Individuals earning less than 5000 LE were more likely to underestimate their risk (aOR = 2.321, *p* = 0.028), suggesting a negative impact of lower income. Furthermore, being overweight or obese significantly increased the likelihood of underestimating CVD risk (aOR = 4.785, *p* = 0.000), indicating that individuals with a higher BMI are more prone to risk underestimation. Lastly, hypertension was a strong predictor, with those having the condition being over three times more likely to underestimate their CVD risk compared to those without hypertension (aOR = 3.558, *p* = 0.000) (Table 4).

**Table 4:** Multivariate logistic regression analysis of factors associated with cardiovascular risk underestimation (*n* = 390)

**Factors predicting underestimation of CVD risk**	**B**	**SE**	**Wald**	**df**	***P* value**	**aOR**	**95% C.I. for EXP (B)**	
							**Lower**	**Upper**
**Age of participants** (years)	0.381	0.059	41.376	1	0.000^*^	1.464	1.303	1.644
**Marital status** (unmarried^a^) **Married**	2.676	1.191	5.049	1	0.025^*^	14.531	1.408	149.99
**Monthly income (LE)** (≥ 5000^a^) ** < 5000**	0.842	0.382	4.858	1	0.028^*^	2.321	1.09	4.909
**BMI** (Underweight, or normal weight^a^) **Overweight, or obese**	1.566	0.466	11.310	1	0.000^*^	4.785	1.922	11.916
**Hypertension** (No^a^) **Yes**	1.269	0.284	20.037	1	0.000^*^	3.558	2.041	6.203

A backward LR Multiple Logistic Regression model was applied. Multicollinearity and interaction items were checked but not found

The model fitness is x^2^ = 111.627, df = 5, *P* =  < 0.001. Hosmer-Lemeshow test = 6.987, df = 8 with *p* = 0.538), classification table (overall correctly classified percentage = 79.5%) indicates a good model

^*^Statistically significant at *p* < 0.05

^a^Refers to reference group

## Discussion

The current study is the first to examine the accuracy of self-perceived CVD risk in perimenopausal and postmenopausal women in Egypt. The high prevalence of obesity, coupled with the substantial burden of hypertension and diabetes in our study underscores the critical importance of investigating CVD risk underestimation in this population. These established CVD risk factors highlight a population at heightened vulnerability, making this study a crucial first step in developing effective prevention strategies. In this study, 44.3% of participants had a moderate or high CVD risk, and a lower proportion of women perceived their CVD risk as moderate or high (27.7%). A previous study conducted in Malaysia [16] revealed that 65.7% of the examined menopausal women had moderate or high CVD risk, and that 91.7% of them perceived their CVD risk as moderate or high. The higher proportions of both predicted and self-perceived CVD risk in Malaysia may be attributed to the study setting, as it was conducted in a primary health clinic. Although the participants had not been previously diagnosed with CVD, they may have had other risk factors or medical conditions that increased their CVD risk. Additionally, being patients in a healthcare setting might have heightened their awareness and perception of CVD risk. The lower proportion of self-perceived moderate or high CVD risk in our study could be explained by the general belief of women that CVD is an almost exclusively male condition [28]. Also, it could be explained by the women's belief that their gender effect could sustain cardio protection even in menopause [16].

The accuracy rate of self-perceived CVD risk among our participants was substantially higher than that reported in studies conducted in Malaysia [16] and Berlin [29] (68.2%, 18.1%, and 41.35%, respectively). Conversely, the proportion of women who underestimated their CVD risk in our study (24.1%) was approximately half that of the Malaysian and Berlin cohorts (48.7% and 48.6%, respectively). The Malaysian study concluded that misconceptions appear to be common among primary care patients and that this could influence CVD risk perception among the studied women. The authors of the Berlin study, in which approximately two-thirds of the women studied were jobless, concluded that the lack of routine and social integration provided by work life would have influenced their CVD risk perception.

In our study, a higher proportion of women underestimated their CVD risk compared to women who overestimated it (24.1%, 7.7%, respectively), and this ratio was twice the ratio (48.7%, 33.2%, respectively) in the study by Nazri et al. [16]. These findings may be attributed to differences in study populations. While our study focused on university employees and workers with no history of CVD, Nazri et al.'s participants were recruited from a primary care clinic. This suggests that women seeking healthcare services may have a heightened awareness of their CVD risk compared to a generally healthy population like university employees or workers. Consequently, the over-optimism regarding CVD risk observed in our study might be more pronounced due to the absence of pre-existing health concerns among participants.

In the current study, a higher proportion of women in the high-risk group underestimated their risk of CVD compared to women in the moderate-risk group (93.3%, 50.6%, respectively), which was in agreement with the results of previous studies conducted on populations of both genders [15, 30]. The authors of these studies concluded that individuals with high CVD risk have a high optimism bias. Also, people who underestimate their risk may be less motivated to adopt healthy lifestyle behaviors, resulting in high CVD risk.

In our study, marital status was found to significantly predict CVD risk underestimation. However, caution should be considered while interpreting the predicting ability of marital status due to the small proportion of women representing the “unmarried” category (6.2%), which contributed to a wide confidence interval with a resultantly low precise representation of the population studied. This association might be explored through the "comparative optimism" theory [31], where married women could perceive themselves as less susceptible to negative health outcomes compared to other family members. However, the association between marital status and underestimation of CVD risk observed in our study warrants further investigation to explore how Egyptian cultural factors might influence this association.

Notably, while education and awareness of CVD risk factors were initially significant, they were not included in the final predictive model. This, along with the inability of numeracy and HL to predict risk underestimation, highlights the potential influence of cultural factors in Egypt. Cultural beliefs may discourage acknowledging potential health risks, leading to a tendency to avoid negative confessions about health.

The ability of hypertension to significantly predict CVD risk underestimation in our study agreed with the results of previous studies conducted on populations from both genders [30, 32]. Lack of strong policies for communicating CVD risks can lead to patients not receiving accurate and timely information about their risk, resulting in an underestimation of CVD risk [30].

Our finding that older age predicts an underestimation of CVD risk aligns with prior research in menopausal populations [16], various age groups [14, 29], and adults of both genders [32]. The optimistic bias theory, which suggests that people underestimate future risk due to the absence of current disease [33], may have contributed to this pattern. However, a previous study concluded that this underestimation primarily stems from a consistent misperception of how CVD risk escalates with age rather than simply an optimistic bias. As CVD risk factors accumulate over time, leading to a substantial increase in overall risk, women's failure to recognize this trend can result in a significant miscalculation of their personal risk profile [29].

The results regarding the predicting ability of BMI in our study were inconsistent with the results of a previous study concluded that individuals with a high BMI have a lower risk underestimation [32]. Variation in results could be explained by the high likelihood of obesity as a personal characteristic that directly affects self-image and interferes with an accurate self-perception of risk [34].

Our finding regarding the ability of lower income to significantly predict CVD risk underestimation aligns with the established literature linking some socioeconomic features, including income, to a lack of awareness about the increased risk and a consequent subjective CVD risk underestimation [14]. The adverse impact of lower income on CVD risk perception might be influenced by limited access to quality healthcare, occupational stressors, or reduced social support. Further research is needed to elucidate these complex relationships and identify specific mechanisms by which lower income contributes to this underestimated risk perception.

### Limitations

This study has some limitations. First, the cross-sectional nature of our study design precludes the establishment of causal relationships between the identified predictors and the underestimation of CVD risk. Second, the sample used in the present study was restricted to the employees and workers at Suez Canal University; therefore, our findings should be extrapolated with caution to non-working women or those working in other jobs. Third, self-reporting was used to determine variables associated with and predicting CVD risk perception, with the possibility of social desirability bias.

## Conclusions

About two-thirds of our study participants accurately perceived their CVD risk. Among those who misperceived their risk, more than three-quarters underestimated it. Marital status, old age, low income, high BMI, and hypertension were strong predictors of CVD risk underestimation. Education, awareness of CVD risk factors, numeracy, and HL didn’t predict CVD risk underestimation. The identification of these predictors underscores the importance of targeted public health interventions and education to improve risk perception accuracy, particularly among vulnerable groups such as older adults, married individuals, those with lower socioeconomic status, higher BMI, and hypertension. Addressing these disparities is crucial for preventing the potential underestimation of CVD risk that could lead to inadequate preventive measures or delays in seeking appropriate medical care.

## Data Availability

The data used in the present study will be made available upon reasonable request from the corresponding author.

## Abbreviations

aOR: Adjusted Odds Ratio
BMI: Body Mass Index
CVD: Cardiovascular Diseases
HL: Health Literacy
HLQ-EU-16: The 16-item Short Version of the European Consortium for Health Literacy Questionnaire
HTN: Hypertension
LR: Likelihood Ratio
PSS: Perceived Stress Scale
SD: Standard deviation
SE: Standard error
SNS: Subjective Numeracy Scale
SPSS: Statistical Package for the Social Sciences
WHO: World Health Organization
